# Intraprocedural activated clotting time and heparin dosage in pulsed field ablation of paroxysmal atrial fibrillation

**DOI:** 10.3389/fcvm.2025.1501716

**Published:** 2025-02-24

**Authors:** Chengming Ma, Xianjie Xiao, Qian Chen, Wenwen Li, Zhongzhen Wang, Shiyu Dai, Yuanjun Sun, Yunlong Xia, Lianjun Gao, Xiaomeng Yin

**Affiliations:** ^1^ Department of Cardiology, Institute of Cardiovascular Diseases, First Affiliated Hospital of Dalian Medical University, Dalian, China; ^2^ Department of Graduate School, Dalian Medical University, Dalian, China; ^3^ Department of Intensive Care Unit, First Affiliated Hospital of Dalian Medical University, Dalian, China

**Keywords:** radiofrequency catheter ablation, pulsed field ablation, activated clotting time, atrial fibrillation, anticoagulant

## Abstract

**Aims:**

Whether the intraprocedural anticoagulation regimen and activated clotting time (ACT) in pulsed field ablation (PFA) for atrial fibrillation (AF) are the same as those for radiofrequency catheter ablation (RFCA) is currently unknown.

**Methods and results:**

Our retrospective study included 51 paroxysmal AF patients who underwent PFA (PFA group) and were matched with paroxysmal AF patients who underwent RFCA. Nearest-neighbor propensity score matching was performed at a 1:1 ratio (no tolerance to anticoagulant regimens and a tolerance of 0.02 on the CHA_2_DS_2_-VASc score, left atrial diameter, and left ventricular ejection fraction). Compared with the RFCA group, the PFA group had a significantly shorter procedure time but a longer fluoroscopy time. In both groups, an initial heparin dose of 110 U/kg was given. The 30-min ACT in the PFA group (240 ± 95.5 s) was shorter than that in the RFCA group (294.4 ± 82.3 s, *P* = 0.003). The 60-, 90-, and 120-min ACTs were significantly longer in the PFA group. The percentage of 30 min-ACTs in the therapeutic range in the RFCA group (33.3%) was greater than that in the PFA group (15.7%, *P* = 0.038). The time to achieve the target ACT was longer in the PFA group. There were no differences in the incidence of periprocedural thromboembolism or bleeding events between the two groups.

**Conclusions:**

Compared with RFCA, PFA was associated with longer intraprocedural ACTs, shorter initial ACTs, fewer initial ACTs in the therapeutic range, and longer times to achieve the target ACT.

## 1 Introduction

Atrial fibrillation (AF) is the most common type of arrhythmia worldwide, and its incidence and prevalence are increasing (1). AF is associated with a 2.4-fold increased risk of stroke and high healthcare costs and expenses for patients, according to previous studies (2). Moreover, the increased risk of heart failure in patients with AF also contributes to the high healthcare burden.

Currently, oral anticoagulants (OACs) and catheter ablation (CA) are the main treatments for patients with AF. OAC is the essential treatment for AF and has been shown to decrease the lifetime risk of stroke (3). Non-vitamin K antagonist oral anticoagulants (NOACs) have become the main drugs used to treat patients with nonvalvular AF. Numerous randomized controlled trials (RCTs) and research involving large registries have revealed that CA is more effective than antiarrhythmic drugs in maintaining sinus rhythm (4, 5). Radiofrequency (RF) has become the main energy source for CA since the pulmonary vein was recognized to play a dominant role in AF in 1998. Cryoablation is an alternative for PVI. Cryoablation and RF ablation are comparable in terms of their effects, degrees of cellular damage, inflammatory response, and thromboembolic risk (6, 7). Notably, patients who undergo CA procedures for AF are at increased risk of stroke and thromboembolism. Periprocedural thromboembolic events (1.0%) and asymptomatic cerebral embolism (5%–15%) are notable complications and are sometimes life-threatening. Therefore, periprocedural anticoagulant management is essential to reduce the risk of thromboembolism, facilitate hemostasis and prevent intra- and postoperative bleeding. Uninterrupted anticoagulation with unfractionated heparin (UFH) to maintain the activated clotting time (ACT) in the safe range during the procedure is the current consensus. Most evidence on UFH and the intraprocedural target ACT (300–350 s) during ablation procedures for AF is derived from studies involving patients undergoing VKA and RFCA procedures (8).

Currently, pulsed field ablation (PFA) is another promising method for PVI in the treatment of AF (9). Unlike RF ablation or cryoablation, PFA is a nonthermal ablation procedure that causes irreversible and selective cardiac electroporation. PFA may not result in collateral damage to noncardiac tissues (10, 11). During PFA, electrical pulses delivered to cardiac cells disrupt the integrity of the cell membrane, causing cell death and replacement fibrosis. Currently, there are few early preclinical and clinical studies supporting the implementation of PFA in clinical practice. Whether PFA is better suited for periprocedural management of CA in patients with AF is unknown because of limited evidence. Whether intraprocedural anticoagulation management and periprocedural thromboembolic risk differs between PFA and RFCA are unknown. Currently, the protocol for intraprocedural anticoagulation management for PFA is the same as that for RFCA. It is uncertain whether the current intraprocedural UFH dosing regimen and target ACT values (300–350 s) are appropriate for PFA. Nevertheless, few studies focusing on the heparin dosage in AF patients who undergo PFA have been reported. We hypothesized that the intraprocedural anticoagulation management and periprocedural thromboembolic risk of PFA would differ from those of RFCA.

We compared the intraprocedural ACTs, time required for the ACT to reach the therapeutic range, actual percentages of measurements at the target ACT, heparin dosage, and incidence of periprocedural thromboembolic and bleeding events between AF patients who underwent PFA and those who underwent RFCA to determine whether the current intraprocedural heparin dosing regimen is appropriate for PFA.

## 2 Methods

### 2.1 Study design

This study was a retrospective, single-center clinical trial involving patients with paroxysmal AF who underwent PFA and RFCA in our center from 1/02/2023–30/06/2024. This trial was approved by the Ethics Committee of the First Affiliated Hospital of Dalian Medical University.

### 2.2 Study subjects

**Figure 1:**
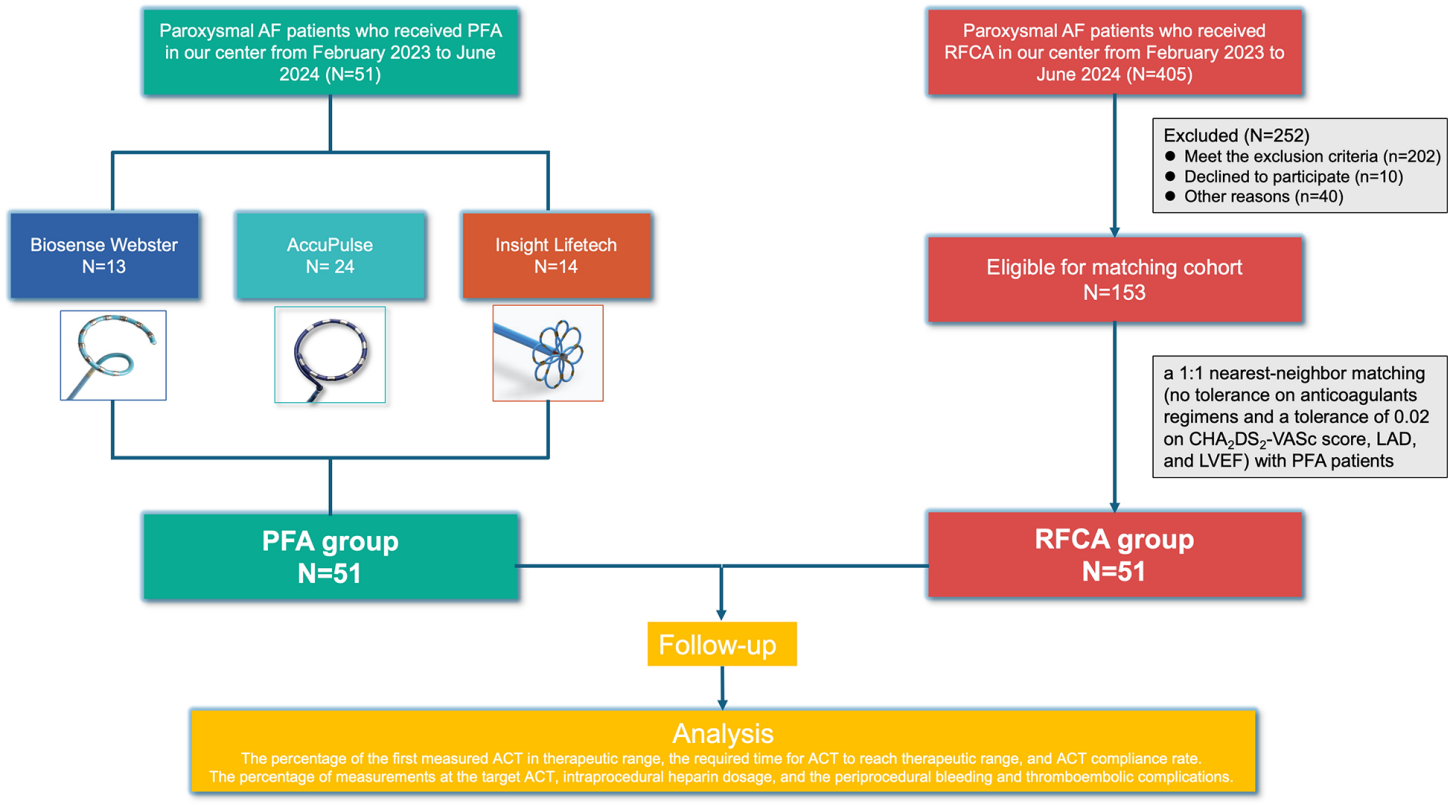
Flow chart of the study.

Patients with paroxysmal AF who underwent PFA and RFCA at our center from 1/02/2023 to 30/06/2024 were retrospectively included. Patients with a history of cardiac ablation, cardiac surgery, or cardiovascular implantable electronic devices; severe hepatic/renal insufficiency; cerebrovascular disease within the last 3 months (including stroke and transient ischemic attack); or contraindications to anticoagulants were excluded. Patients with severe cardiac dysfunction [a left ventricular ejection fraction (LVEF) ≤40% or NYHA cardiac function grade III–IV] or a left atrial diameter (LAD) ≥55 mm were also excluded. Patients who refused to participate in this trial and were lost to follow-up were not included (Figure 1).

The patients in the PFA group were collected from the three prospective studies conducted in our center to evaluate the safety and effectiveness of PFA in paroxysmal AF: study A: the AFIRE study (a prospective, multicenter, single arm study with performance goals designed to evaluate the safety and effectiveness of a multielectrode circular IRE catheter and multichannel IRE generator in the treatment of paroxysmal AF, NCT05552963); study B: the PF-Beat-AF Trial [a study of pulse field ablation (PFA) for the treatment of paroxysmal atrial fibrillation, AccuPulse Medical Technology]; and study C: the Comparison of PFA vs. RFA in Patients with Symptomatic Paroxysmal Atrial Fibrillation (NCT06014996, Insight LifeTech). Patients in the RFCA group underwent RFCA at our center at the same time as those in the PFA group underwent PFA. PFA patients were matched 1:1 with RFCA patients via nearest-neighbor propensity score matching with no tolerance of anticoagulant regimens and a tolerance of 0.02 for the CHA2DS2-VASc score, LAD, and LVEF. Aside from the parameters mentioned above, weight, age, hepatic and kidney function, past medical history, date of procedure, and the operator were also considered during the matching process.

### 2.3 Preprocedural anticoagulant

All patients were treated with NOACs preoperatively, and atrial thrombosis was excluded by computed tomography or transesophageal echocardiography of left atrial appendage in female patients with CHA2DS2-VASc scores ≥ 3 and male patients with CHA2DS2-VASc scores ≥ 2 within 48 h before the ablation procedure.

### 2.4 Catheter ablation

The CA procedure was performed under general anesthesia. A bolus of 110 U/kg of heparin was administered immediately after right femoral vein puncture. A decapolar catheter was positioned in the coronary sinus for atrial pacing and signal reference. Transseptal puncture was performed via a modified Brockenbrough technique.

In the PFA group, ablation was performed via the following three specialized PFA generators and PFA catheters: (1) the Multi-Channel Irreversible Electroporation (IRE) generator and Multi-Channel Circular IRE catheter (Biosense Webster, Irvine, USA); (2) the AccuPulse PFA generator and circular-shaped PFA catheter [AccuPulse Medical Technology (Suzhou) Co. Ltd.; Jiangsu, China]; and (3) the PFA generator (Insight Lifetech, Shenzhen, China), a proprietary lotos-shaped PFA catheter (LotosPFA, Insight Lifetech), and a customized steerable sheath to navigate and position the PFA catheter.

In the RFCA group, a PV mapping catheter (Pentaray NAV ECO High Density Mapping Catheter, Biosense Webster, Irvine, USA, or Advisor HD Grid Mapping Catheter, Abbott Laboratories, Chicago, IL) and a saline-irrigated ablation catheter (Thermocool SMART TOUCH SF, Biosense Webster, Irvine, USA., or TactiCath Contact Force Ablation Catheter) were used for mapping and ablation via the CARTO 3 V6 electroanatomic mapping system (Biosense Webster, Irvine, USA) or Ensite Precision Cardiac Mapping System (Abbott Laboratories, Chicago, IL).

The final stage of the CA procedure was complete electrical isolation of the PV, which was confirmed by the absence of PV potentials or PV-left atrium conduction; notably, no tachycardia was induced by the electrophysiologic study (EPS).

### 2.5 Intraprocedural heparin administration and ACT monitoring

The initial dosage of heparin was administered immediately after right femoral vein puncture. The amount of supplemental heparin used was determined by the operator to maintain the ACT between 300 and 350 s based on our previous study (12). An additional dose of heparin was not given if the ACT was ≥350 s. If ACT was 150–300 s, a heparin dose of 800 U was added, if ACT < 150 s, a heparin dose of 1,000 U was added. An additional dose of heparin was not given if the ACT reached the target or was ≥300 s. If severe bleeding complications occurred, heparin was discontinued immediately, and then protamine sulfate was administered.

The ACT was measured every 30 min with an optical coagulation analyzer (OCG-102, Wondfo Biotech, Guangzhou, China). ACT compliance was defined as at least one intraprocedural ACT in the therapeutic range. The intraprocedural ACTs at each 30-min interval (30 min-ACT, 60 min-ACT, 90 min-ACT, 120 min-ACT, and 150 min-ACT), average percentage of measurements at the target ACT, percentage of initial ACTs (i.e., 30 min-ACT) in the therapeutic range, time required for the ACT to reach the therapeutic range, intraprocedural heparin dose, ACT compliance rate, and total amount of heparin administered were collected and analyzed.

### 2.6 Follow-up

The incidence rates of periprocedural bleeding and thromboembolic complications were recorded and analyzed. The periprocedural complications were defined as adverse events that occurred within 30 days after the procedure. The primary endpoints were the percentage of initial ACTs in the therapeutic range, time required for the ACT to reach the therapeutic range, and ACT compliance rate. The secondary endpoints were the percentages of measurements at the target ACT, intraprocedural heparin dosage, and periprocedural bleeding and thromboembolic complications.

### 2.7 Statistical analysis

SPSS version 27.0 (SPSS Inc., Chicago, USA) was used for all analyses. Continuous variables are expressed as the mean ± standard deviation (SD) if normally distributed; the median and the 25%–75% interquartile range were used for skewed data. An unpaired t test or one-way analysis of variance was performed for measurement data. For non-normally distributed measurement data, the Mann–Whitney *U* test was performed for comparisons between two groups. The Kruskal‒Wallis *H* test with Bonferroni correction was used for comparisons between multiple groups. For categorical variables, chi-square tests or Fisher's exact tests were used for comparisons between two groups. The ACTs and actual percentages of measurements at the target ACT were compared between the RFCA group and the PFA group. A 2-tailed *P* value < 0.05 indicated statistical significance.

## 3 Results

### 3.1 Baseline characteristics

A total of 102 AF patients [59.9 ± 9.1 years; 58 (56.9%) males] were included in this study. There were 51 patients in the PFA group and 51 patients in the RFCA group. The mean LAD and LVEF were 36.8 ± 3 mm and 59 ± 1.5%, respectively. The mean CHA2DS2-VASc score was 1.4 (0 in 21 (20.6%) patients, 1 in 38 (37.3%) patients, 2 in 24 (23.5%) patients, 3 in 16 (15.7%) patients, 4 in 2 (2%) patients and 5 in 1 (1%) patient). There was no significant difference between the two groups in terms of age, sex, comorbidities, renal function, hemoglobin, concomitant antiplatelet therapy, CHA2DS2-VASc score, left ventricular diameter, etc. Compared with the RFCA group, the PFA group had significantly shorter procedure times (105.8 ± 33.3 min vs. 155.8 ± 40.2 min, *P* < 0.001) but longer fluoroscopy times (16.2 ± 7.5 min vs. 6.7 ± 2 min, *P* = 0.024). The baseline clinical characteristics of the two groups are summarized in Table 1.

**Table 1:** Baseline characteristics of the patients.

	Overall (*n* = 102)	PFA group (*n* = 51)	RFCA group (*n* = 51)	*P*
Age (years)	59.9 ± 9.1	60.1 ± 8.6	59.6 ± 9.7	0.35
Male	58 (56.9%)	28 (54.9%)	30 (58.8%)	0.689
Weight (kg)	75.3 ± 12.3	75.8 ± 12.8	74.7 ± 12.8	0.666
Coronary artery disease	7 (6.9%)	4 (7.8%)	3 (5.9%)	0.999
Hypertension	43 (42.2%)	25 (49%)	18 (35.3%)	0.16
Diabetes mellitus	7 (6.9%)	4 (7.8%)	3 (5.9%)	0.999
Heart failure	0	0	0	-
Stroke/TIAs	0	0	0	-
Ccr (ml/min)	67.4 ± 14.3	67.6 ± 13.9	67.2 ± 14.7	0.89
Hemoglobin (g/L)	141.1 ± 14.4	140.6 ± 15	141.6 ± 13.8	0.707
Thrombocyte (10^9^/L)	215.3 ± 55.8	218.9 ± 55.8	211.6 ± 56.1	0.514
Alanine aminotransferase	24 (17, 31.3)	23 (16, 31)	24 (17, 31.5)	0.361
Aspartate aminotransferase	23 (20, 29.3)	23 (20,29)	24 (20,30)	0.809
CHA_2_DS_2_-VASc score				0.211
0	21 (20.6%)	9 (17.6%)	12 (23.5%)	
1	38 (37.3%)	18 (35.3%)	20 (39.2%)	
2	24 (23.5%)	14 (27.5%)	10 (19.6%)	
3	16 (15.7%)	8 (15.7%)	8 (15.7%)	
4	2 (2%)	2 (3.9%)	0	
5	1 (1%)	0	1 (2%)	
LAD (mm)	36.8 ± 3	36.8 ± 2.7	36.9 ± 3.4	0.896
LVEF (%)	59 ± 1.5	58.8 ± 1.3	59.2 ± 1.6	0.175
Anticoagulants (n)				
Edoxaban	16 (15.7%)	8 (15.7%)	8 (15.7%)	
Rivaroxaban	86 (84.3%)	86 (84.3%)	86 (84.3%)	
Concomitant antiplatelet therapy	0	0	0	-
Procedure time (min)	142.3 ± 44.3	105.8 ± 33.3	155.8 ± 40.2	<0.001
x-ray time (min)	14.6 ± 7.8	16.2 ± 7.5	6.7 ± 2	0.024

### 3.2 Intraprocedural ACTs

**Figure 2:**
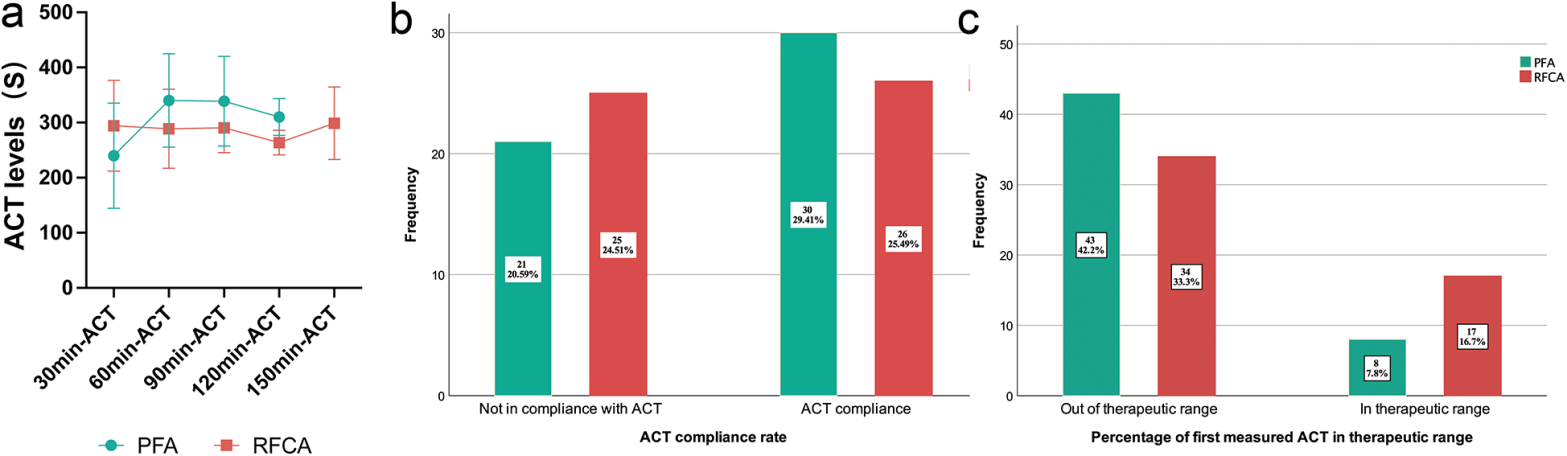
Intraprocedural ACTs of the PFA group and RFCA group. **(a)** shows the intraprocedural ACTs at each 30-minute interval, **(b)** shows the ACT compliance rate of the PFA group and the RFCA group and **(c)** shows the percentage of first measured ACT in the therapeutic range of the PFA group and the RFCA group. The percentages in the bar chart represent the proportion of all patients.

During the RFCA procedure, each patient underwent 2.4 ± 1 ACT measurements, and patients in the PFA group underwent 2.3 ± 1 ACT measurements (*P* = 0.556). The ACTs at 30-min intervals are shown in Figure 2A and Table 2. The 30-min ACT in the PFA group (240 ± 95.5 s) was shorter than that in the RFCA group (294.4 ± 82.3 s, *P* = 0.003). The 60 min-ACT, 90 min-ACT, and 120 min-ACT were significantly longer in the PFA group than in the RFCA group (Table 2). The average percentage of measurements at the target ACT in the PFA group (33% [0,50%]) was greater than that in the RFCA group (20% [0,50%], *P* = 0.565), but the difference was not statistically significant.

**Table 2:** Intraprocedural ACTs.

ACT (s)	Overall (*n* = 102)	PFA group (*n* = 51)	RFCA group (*n* = 51)	Difference (95% CI)	*P*
30 min-ACT	267.2 ± 92.8	240 ± 95.5	294.4 ± 82.3	−54.4 (−89.4 to −19.4)	0.003
60 min-ACT	314.1 ± 82.3	340 ± 84.9	288.9 ± 71.9	51.1 (16.8 to 85.5)	0.004
90 min-ACT	314.1 ± 69	338.8 ± 81.4	290.6 ± 45.2	48.2 (5.8 to 90.5)	0.027
120 min-ACT	288.7 ± 36.8	310.1 ± 33.5	263.6 ± 22.5	46.5 (10.9 to 82.1)	0.015
150 min-ACT	298.6 ± 65.9	-	298.6 ± 65.9		-
Percentage of measurements at the target ACT (%)	25% [0,50%]	33% [0.50%]	20% [0,50%]	0 (0 to 8%)	0.565
Percentage of initial ACTs in therapeutic range (%)	25 (24.5%)	8 (15.7%)	17 (33.3%)	−17.6% (−34% to −1.3%)	0.038
ACT compliance rate (%)	56 (54.9%)	30 (58.8%)	26 (51%)	7.8% (−11.4% to 27.1%)	0.426

There was no significant difference in the ACT compliance rate between the two groups (58.8% in the PFA group vs. 51% in the RFCA group, *P* = 0.426; Figure 2B). However, the percentage of initial ACTs (30 min-ACT) in the therapeutic range in the RFCA group (33.3%) was markedly greater than that in the PFA group (15.7%, *P* = 0.038), indicating that the ACT in the RFCA group reached the target more quickly with the same initial dose of heparin (shown in Figure 2C).

**Figure 3:**
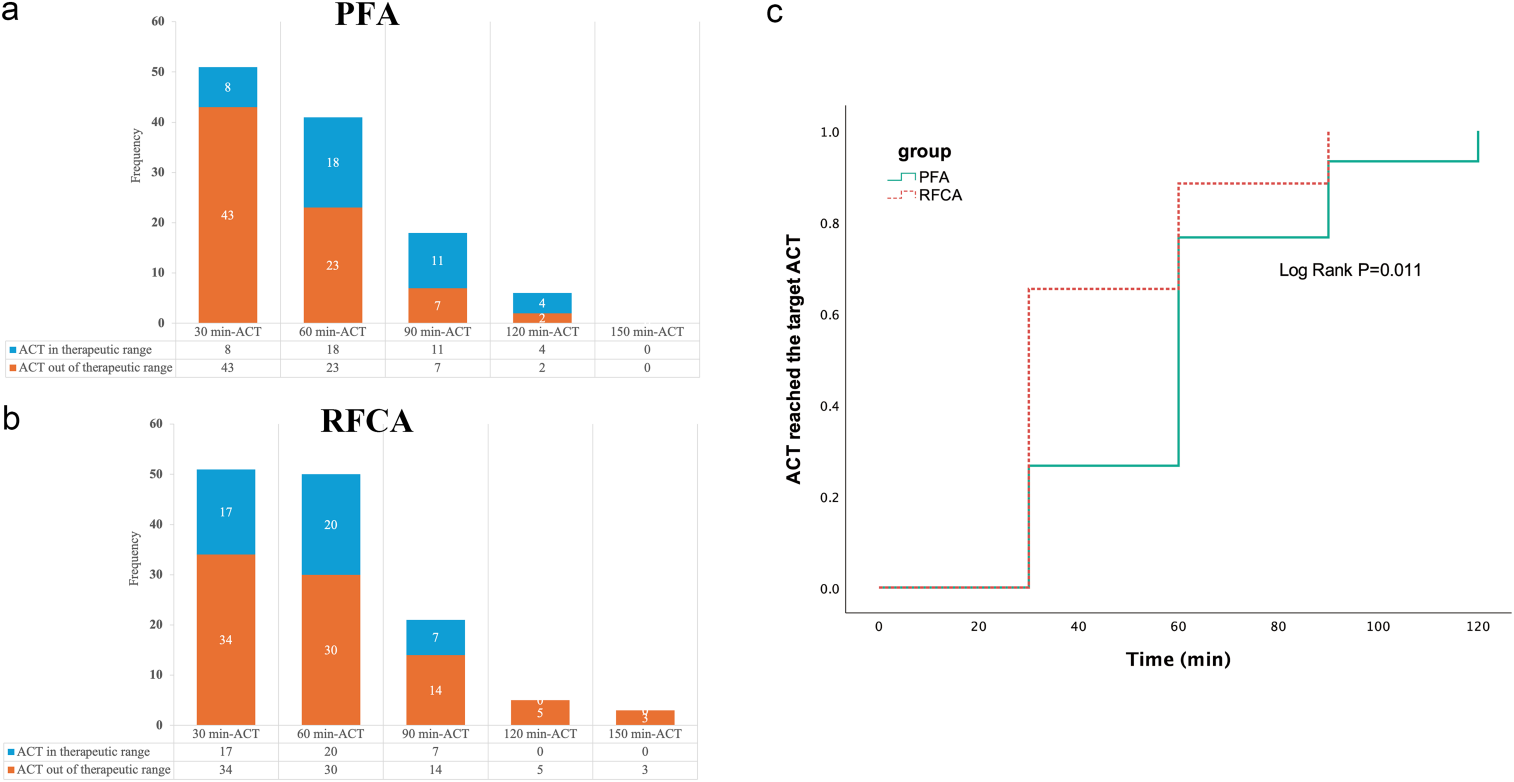
The number of ACT measurements and Kaplan–Meier analysis of the PFA group and RFCA group. **(a)** shows the number of ACT measurements and the percentage of ACTs in or out of the therapeutic range at each 30-minute interval in the PFA group, **(b)** shows the number of ACT measurements and the percentage of ACTs in or out of the therapeutic range at each 30-minute interval in the RFCA group and **(c)** shows that the patients in the PFA group required more time to achieve the target ACT than did the patients in the RFCA group.

Figure 3A,B show the number of ACT measurements and the percentage of ACTs in or out of the therapeutic range throughout the whole procedure. In the PFA group, 10 patients did not undergo a second ACT measurement, as the procedure was already complete. Thirty-one patients underwent two ACT measurements, representing 64.7% (31/51) of all PFA patients. The patients in the PFA group needed more time to achieve the target ACT than did the patients in the RFCA group (Figure 3C).

**Figure 4:**
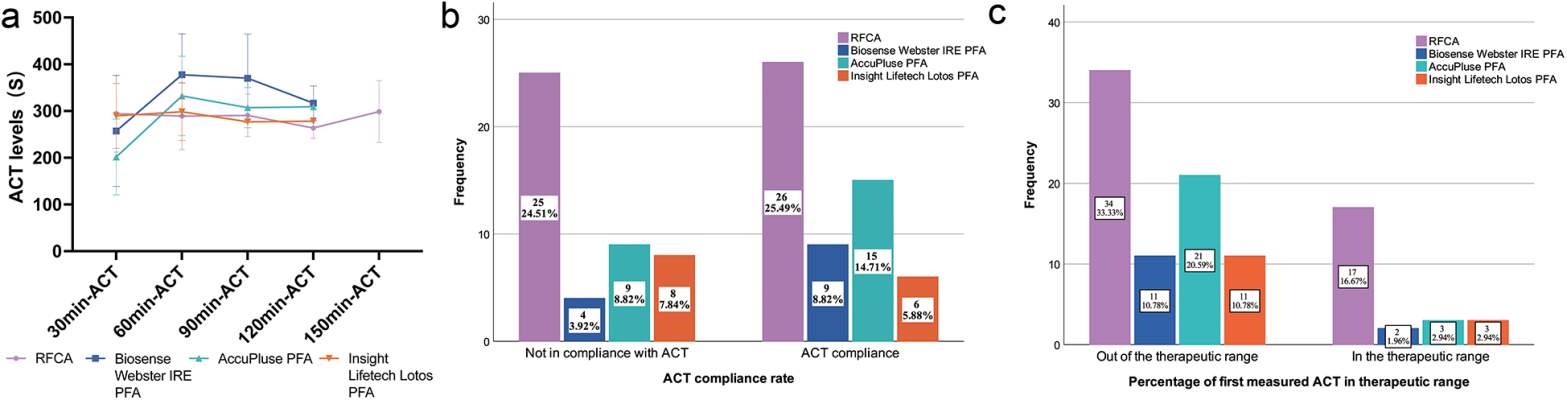
Intraprocedural ACTs from the subgroup analysis of the PFA group. **(a)** shows the intraprocedural ACTs of the four groups at each 30-minute interval, **(b)** shows the ACT compliance rate of the four groups and **(c)** shows the percentage of first measured ACT in the therapeutic range of the four groups. The percentages in the bar chart represent the proportion of all patients.

In the subgroup analysis of the PFA group, no significant differences in 60 min-ACT, 90 min-ACT or 120 min-ACT were detected among A, B, or C, as shown in Table 3 and Figure 4A. No difference in the average percentage of measurements at the target ACT, ACT compliance, or percentage of initial ACTs in the therapeutic range was detected between the types of PFA (Table 3 and Figure 4B,C).

**Table 3:** Subgroup analysis of intraprocedural ACTs in the PFA group.

ACT (s)	*A* (*n* = 13)	*B* (*n* = 24)	*C* (*n* = 14)	*P*	*PA-B*	*PA-C*	*PB-C*
30 min-ACT	257.4 ± 118.6	201.7 ± 81.1	289.5 ± 69.3	0.015	0.076	0.355	0.005
60 min-ACT	377.6 ± 87.5	332.2 ± 84.9	298.4 ± 61.4	0.096	0.128	0.038	0.33
90 min-ACT	370.1 ± 94.5	307.1 ± 42.9	277	0.147			
120 min-ACT	316.8 ± 37.1	309	278	0.663			
Percentage of measurements at the target ACT (%)	25% [0,67%]	33% [0,67%]	0 [0,50%]	0.465	1.000	1.000	0.65
Percentage of initial ACTs in therapeutic range (%)	2 (15.4%)	3 (12.5%)	3 (21.4%)	0.882			
ACT compliance rate (%)	9 (69.2%)	15 (62.5%)	6 (42.9%)	0.354			

### 3.3 Heparin dosage

The required heparin dosage to achieve the target ACT, including the initial, additional, and total dose of heparin, was similar between the PFA group and the RFCA group (Table 4). The proportion of patients (78.4%) who needed an additional dose of heparin in the RFCA group was greater than that in the PFA group (56.9%, *P* = 0.02).

**Table 4:** Intraprocedural heparin.

	Overall (*n* = 102)	PFA group (*n* = 51)	RFCA group (*n* = 51)	Difference (95% CI)	*P*
Initial heparin	8,199 ± 1,538	8,486.3 ± 1,342.7	7,911.8 ± 1,675.4	574.5 (−22 to 1,171)	0.059
Added heparin	2,405.8 ± 1,295.6	2,456.5 ± 1,295.1	2,363.5 ± 1,310.6	103 (−531.9 to 737.9)	0.747
Total heparin	9,826.5 ± 1,934.8	9,888.2 ± 1,802.8	9,764.7 ± 2,074.5	123.5 (−640 to 887.1)	0.749
Numbers of added heparin	69 (67.6%)	29 (56.9%)	40 (78.4%)	−21.6% (−39.2% to −3.9%)	0.02

### 3.4 Bleeding and thromboembolic complications

There was no significant difference in the incidence of bleeding or thromboembolic complications between the two groups (Table 5). Two inguinal hematomas were observed in the PFA group, and three inguinal hematomas (including one arteriovenous fistula) were observed in the RFCA group. No hematuria/hemoglobinuria was detected in the two groups.

**Table 5:** Bleeding and thromboembolic complications.

Complications (*n*)	PFA group	RFCA group	*P*
Thromboembolic complications (*n*)	0 (0)	0 (0)	N/A
Stroke/TIAs	0 (0)	0 (0)	N/A
DVT	0 (0)	0 (0)	N/A
PE	0 (0)	0 (0)	N/A
Bleeding complications (*n*)	2 (3.9%)	3 (5.9%)	0.843
Cardiac tamponade	0 (0)	0 (0)	N/A
Pericardial effusion	0 (0)	0 (0)	N/A
Retroperitoneal hemorrhage	0 (0)	0 (0)	N/A
Hemoglobin drop ≥ 4 g/dl	0 (0)	0 (0)	N/A
Blood transfusion required	0 (0)	0 (0%)	1.000
Inguinal hematoma	2 (3.9%)	3 (5.9%)	0.843
Hematuria/Hemoglobinuria	0 (0)	0 (0)	N/A

## 4 Discussion

In the present study, we retrospectively collected and analyzed the intraprocedural ACTs, periprocedural bleeding and thromboembolic complication rates, and heparin dosages in paroxysmal AF patients who underwent PFA at our center. The significant findings of our study were as follows: (1) PFA was more effective in terms of ablation and a longer fluoroscopy time, with no significant difference between the types of PFA catheters; (2) compared with RFCA, PFA was associated with longer intraprocedural ACTs, shorter initial ACTs, and fewer initial ACTs in the therapeutic range, independent of the type of PFA catheter; (3) the time to achieve the target ACT was longer in patients who underwent PFA than in those who underwent RFCA; and (4) the additional dose of heparin needed to achieve and maintain the target ACT was similar in patients who underwent PFA to those in patients who underwent RFCA. To our knowledge, this is the first study investigating the differences between PFA and RFCA in terms of intraprocedural ACTs and periprocedural bleeding and thromboembolic complication rates.

PVI has been considered essential in CA for AF since PV potentials have been confirmed to trigger paroxysmal AF and the monitoring of PV potentials has been proven paramount (13). Thermal ablation, predominantly RFCA, has been the main treatment method for CA of AF in the last two decades. However, some thermal-related complications, such as atrioesophageal fistula, esophageal perforation, and adjunctive nerve injury, may be severe or immediately life-threatening and may require emergency management. PFA is a novel and promising procedure (14, 15). Although data for PFA are still limited, the existing evidence indicates that the incidence of adverse extracardiac effects is expected to be significantly lower due to electroporation into cardiomyocytes (16).

Stroke and asymptomatic acute cerebral lesions are serious periprocedural thromboembolic events with an incidence of 0.1%–0.5% and 5%–30%, respectively, that cannot be ignored and have lifelong consequences (17). The incidence of MRI-detected brain lesions after thermal ablation of AF was nearly 30% (18, 19). In contrast to the significant decrease in the incidence of adjunctive tissue damage, the incidence of periprocedural thromboembolic events associated with PFA did not decrease significantly (20). The analysis of the neurological assessment subgroup in the ADVENT trial, which compared the cerebral impact of thermal and PFA ablation in treating PAF, revealed a comparable incidence of silent cerebral events (SCEs) and silent cerebral lesions (SCLs) following PVI ablation between PFA and RFCA (21). Therefore, safe and adequate intraprocedural anticoagulation management is also essential for PFA of AF. However, there is no consensus on the intraprocedural heparin dosing regimen and the target ACT for PFA, and few studies have focused on this issue.

The present intraprocedural anticoagulation protocol for PFA procedures is in accordance with the established guidelines for RFCA (22). The intraprocedural target ACT for PFA maybe clear (23). However, the appropriateness of current intraprocedural heparin regimens for PFA and whether intraprocedural ACTs are dependent on the type of PFA are still unclear. Different energy generates different lesions, consequently leading to diverse formation of microthrombi (24–26). In our study, we found that the PFA was associated with a longer intraprocedural ACT. However, the initial ACT and number of initial ACTs in the therapeutic range in PFA patients were lower than those in RFCA patients, and the time required to achieve the target ACT was longer in PFA than in RFCA. This could be attributed to real-time heparinized saline irrigation shortening the time to reach the target ACT during RFCA. In the later stages of RFCA, the formation of thrombi and microthrombi may have resulted in less pronounced increases in the ACT. The prolonged subsequent intraprocedural ACT observed in the PFA cohort may be attributed to the higher additional heparin bolus administered. Furthermore, the intraprocedural ACTs were found to be correlated with the heparin dosing regimens. Previous studies have indicated that a modified heparin dosing regimen may improve the ACT compliance rate and the required time to reach the target ACT (27–29). The ACT compliance rate in these modified regimens was higher than that observed in the present study, indicating that a novel heparin dosing regimen is a crucial component of the PFA.

In our study, three PFA catheters that were shaped differently were used: two circular shapes (tending to adhere to the thrombus) and one lotos shape, the latter of which was flexible and resulted in better catheter adherence and energy delivery. We also found that the PFA catheter used for saline irrigation in study A (Biosense Webster IRE Catheter) may have prolonged the ACT. PFA catheters are available in a variety of shapes and irrigated configurations, which result in different intraprocedural ACTs. As PFA becomes more widely used in clinical practice and post-market applications, it is essential to consider the impact of this variability on periprocedural anticoagulation management.

In our study, although the total amount of heparin administered was not significantly different between the two groups, the number of additional units of heparin administered to the PFA group was fewer than that administered to the RFCA group. PFA is a short procedure and may have been completed before an additional dose of heparin was administered. The discrepancy in the total heparin dosage was attributable to the necessity of a 30-minute observation period during clinical studies in the PFA cohort, during which additional heparin was administered to mitigate the risk of embolization. Thus, we suggest a higher initial dose of heparin for PFA. Moreover, according to the latest study of 337 patients undergoing PFA for AF, PFA may redefine the blanking period of AF ablation (30). This offer more benefits to AF patients, such as the reduced necessity for post-procedure anticoagulation therapy.

This study has several limitations. This was a single-center retrospective study with a small sample of patients. Due to the low incidence of hemorrhagic event and no thrombotic complication, it was underpowered to detect differences in periprocedural bleeding or thromboembolic complication rates. The incidence of silent cerebral ischemia after ablation was not evaluated. Multicenter prospective RCTs with a larger sample of patients and MRI-detected SCLs are needed to verify this hypothesis. Moreover, few measurements were at the target ACT in this study. This study exclusively examined intraprocedural ACTs for PFA and RFCA, and did not evaluate intraprocedural ACT management and anticoagulation strategies for other emerging technologies, such as cryoablation or laser ablation. This was due to the limited procedural volume and technical constraints. In addition, the study concentrated on the peri-procedural management, and a long-term follow-up was not conducted.

## 5 Conclusion

Compared with RFCA, PFA was associated with longer intraprocedural ACTs, shorter initial ACTs, fewer initial ACTs in the therapeutic range, and longer times to achieve the target ACT.

## Data Availability

The original contributions presented in the study are included in the article/Supplementary Material, further inquiries can be directed to the corresponding authors.
